# Changes in Back Pain Scores after Bariatric Surgery in Obese Patients: A Systematic Review and Meta-Analysis

**DOI:** 10.3390/jcm10071443

**Published:** 2021-04-01

**Authors:** Froukje W. Koremans, Xiaolong Chen, Abhirup Das, Ashish D. Diwan

**Affiliations:** 1 Amsterdam UMC, Vrije Universiteit Amsterdam, Surgery, Amsterdam Trauma Surgery, De Boelelaan, 1117 Amsterdam, The Netherlands; F.W.Koremans@olvg.nl; 2 Spine Labs, St. George and Sutherland Clinical School, University of New South Wales, Kogarah, NSW 2217, Australia; xiaolong.chen1@unsw.edu.au (X.C.); a.diwan@unsw.edu.au (A.D.D.); 3 Spine Service, St. George Hospital, Kogarah, NSW 2217, Australia

**Keywords:** low back pain, bariatric surgery, obesity, meta-analysis, meta-regression

## Abstract

Bariatric surgery produces significant and quantifiable reductions in back pain. However, there is a lack of information on the association of weight changes after bariatric surgery with changes in pain score. We aim to evaluate the impact of bariatric surgery on back pain in obese patients and to address the association between changes in body mass index (BMI) and pain score. In obese patients eligible for bariatric surgery, the changes in pre- and post-operative pain scores, assessed by the Numeric Rating Pain Scale (NPS) or Visual Analogue Scale (VAS), were considered as primary outcomes. Mean difference (MD) and their 95% confidence intervals (CI) were evaluated. Eight cohort studies were included in the analysis of 298 obese patients undergoing bariatric surgery. All studies showed a reduction in back pain, with a mean change of −2.9 points in NPS and of −3.8 cm in VAS. There was a significant reduction in back pain (NPS: (MD = −3.49) (95% CI = −3.86, −3.12); VAS: MD = −3.75, (95% CI = −4.13, −3.37)) and BMI (MD = −12.93, (95% CI = −13.61, −12.24)) following bariatric surgery. No significant relationship between BMI change and decrease in clinical scores could be established. However, it was evident that bariatric surgery had a significant effect on back pain scores in severely obese patients. Ideally, a prospective study including spinal imaging, inflammatory markers, a longer follow-up period, and larger study groups with a randomized control group needs to be performed.

## 1. Introduction

Obesity is a growing health problem worldwide. The WHO reported that about 1.9 billion adults were overweight and 600 million adults were obese in 2016, numbers which continue to rise over the years [1]. Obesity is defined as an excessive or abnormal fat accumulation and is calculated by the body mass index (BMI). It is a complex and multifactorial condition, with a great risk for the patient’s health. A BMI above 25 mg/kg^2^ is defined as overweight, and a BMI of 30 or more as obese. Obesity is associated with an increased risk for type 2 diabetes, hypertension, cardiovascular diseases, cancer and musculoskeletal diseases, including low back pain (LBP) [2,3,4]. Among obese patients, the prevalence of LBP ranges from 22% to 68.1% [5,6]. According to the American Obesity Association (AOA), LBP is prevalent in nearly one-third of Americans classified as obese. Obesity was previously reported as an important cause of LBP [7,8,9,10]. Therefore, understanding the impact of weight control on LBP is required.

Over the past decade, bariatric surgery has emerged as an attractive, evidence-based option that offers significant and durable weight loss and improved health outcomes for those with clinically severe obesity. American Society for Metabolic and Bariatric Surgery guidelines suggest the position statement on consensus for BMI (35 to 40 without comorbidities or 30 to 35 with significant comorbidities) as an indication for bariatric surgery [11,12]. Besides weight reduction, bariatric surgery also produces significant and quantifiable reductions in back pain [13]. We hypothesize that the resulting consequence of this is a cascade of weight loss initiated by bariatric surgery, which in turn can beneficially affect the patient’s back pain. A previously published systematic review [13] and meta-analysis [14] have reported that bariatric surgery in severely obese patients decreases the intensity of LBP and also decreases disability due to secondary back problems. However, there is a lack of information on the association of weight changes following bariatric surgery with pain score change.

In order to understand the clinical significance of LBP after bariatric surgery, the main objectives of the present review are (1) to evaluate the impact of bariatric surgery on the weight of severely obese patients; (2) to identify the effect of bariatric surgery on the pain score and secondary outcomes (disability and disc space height); and (3) to evaluate the association of weight change with pain score or disability score change if any.

## 2. Materials and Methods

### 2.1. Search Strategies

The literature was searched in accordance with preferred reporting information for systematic reviews and meta-analyses (PRISMA) guidelines [15]. The online databases EMBASE, MEDLINE, PubMed and Cochrane Central Register of Controlled Trials were searched to identify all relevant studies published in English between 1966 and October 2020. The search included the following terms: “bariatrics”, “obesity”, “gastric bypass”, “gastric sleeve”, “Roux-en-Y”, “RYGB”, “sleeve gastrectomy”, “adjustable gastric band” and “back pain”, with appropriate combinations of operators “AND”, “OR”, and “NOT” as described in Supplemental Table S1. Additional references were assessed using the reference lists of relevant studies. The review protocols are registered on PROSPERO (International Prospective Register of Systematic Reviews number, CRD42021189111).

### 2.2. Inclusion Criteria

(1) Randomized controlled trials (RCT) and observational studies of any bariatric surgery (bariatrics, gastric bypass, gastric sleeve, Roux-en-Y, RYGB, sleeve gastrectomy, or adjustable gastric band).
(2) Studies which enrolled adult obese patients with a BMI > 40 kg/m^2^, or a BMI ≥ 35kg/m^2^ with comorbidities, undergoing primary bariatric surgery.
(3) Studies which reported the pain intensity change of the lower back with the Numeric Rating Pain Scale (NPS) or Visual Analogue Scale (VAS) before and after bariatric surgery.
(4) Studies which reported a follow-up of at least 3 months.

### 2.3. Exclusion Criteria

Meta-analyses, systematic reviews, editorials, in vitro biomechanical studies, and studies looking into LBP caused by pathological entities were excluded.

### 2.4. Types of Outcomes Measures

The following outcomes measures were assessed in this review:

(1) Primary outcome: the change in the pain intensity score of the LBP before and after bariatric surgery, as measured in VAS or NPS, and the change in BMI before and after bariatric surgery.
(2) Secondary outcomes: the change in disc space height, back-specific disability questionnaires (Roland–Morris score, Oswestry Disability Index (ODI), and Waddell Disability Index) and pain pressure threshold (PPT) before and after bariatric surgery; the association between the back-specific disability questionnaires (Roland–Morris score or Oswestry Disability Index (ODI) or Waddell Disability Index) and BMI changes following bariatric surgery for severely obese patients.

### 2.5. Selection of Studies

Two reviewers (FWK and XLC) screened the titles and abstracts following the inclusion and exclusion criteria. In all of the potential eligible studies, the full text was reviewed. When consensus could not be reached between the reviewers, a third reviewer (AD) was consulted to resolve the disagreement.

### 2.6. Data Extraction

Two reviewers (FWK and XLC) extracted data independently. The reviewers collected the following data: methods (first author’s name, publication year, study design, sample size, inclusion and exclusion criteria, mean duration of follow-up), participants (number of participants, age, gender), interventions (surgical procedure), and outcomes (for each primary outcome: preoperative and post-operative pain intensity scores and BMI; secondary outcome: back-specific disability scores).

### 2.7. Risk Bias Assessment

The Newcastle–Ottawa Scale (NOS) was used to assess the methodological quality of the included observational studies [16]. The NOS tool judges an article on three domains: (1) selection of the group, (2) comparability and (3) assessment of the outcomes. The score of a study can range from 0 to 9, where a study with score of 7 or higher will be categorized as low risk. A sensitivity analysis was conducted to assess the impact of including studies with a high overall risk of bias. The assessments were discussed with a second researcher (XLC), and disagreements were resolved by the third reviewer (AD).

### 2.8. Statistical Analysis

Mean difference (MD) and standard deviation (SD) were calculated. To calculate MD, the mean change for the post-operative data was subtracted from the mean change for pre-operative data. The SD was generated by dividing the standard deviations by the square root of the study population. The chi-squared (I^2^) statistic was used to measure heterogeneity among the trials. I^2^ < 50% implied homogeneity, and the analysis included a random-effects model by the DerSimonian–Laird method. I^2^ > 50% indicated heterogeneity and, consequently, a fixed-effects model was used according to the Mantel–Haenszel method. We conducted subgroup analysis and sensitivity analysis to assess the impact of heterogeneity. MD and 95% confidence intervals (CI) were reported. A forest plot was used to calculate the results. Publication bias was assessed by funnel plot symmetry using the Begg–Mazumdar test. The statistical significance was set at 5% (α = 0.05).

Meta-regression was used for moderator analyses because it reduces the probability of type I error by computing concurrent estimates of independent effects by multiple moderators on the variation in effect size across trials. To calculate the association of mean pain change with BMI change, we included BMI change as a predictor in a meta-regression analysis.

Finally, this meta-analysis was performed according to the Meta-analysis of Observational Studies in Epidemiology group recommendations for improving the quality of reporting of meta-analyses of clinical observational studies, respectively [17,18]. STATA software (Release 15, StataCorp LLC, College Station, TX, USA) was used for the statistical analyses.

### 2.9. Quality Assessment

The Grading of Recommendations Assessment, Development and Evaluation (GRADE) system was used to evaluate the levels of evidence, the quality of assessment and the results from data extraction [19]. The quality was rated as “very low”, “low”, “moderate” or “high” (Supplemental Table S2).

## 3. Results

### 3.1. Study Selection

**Figure 1:**
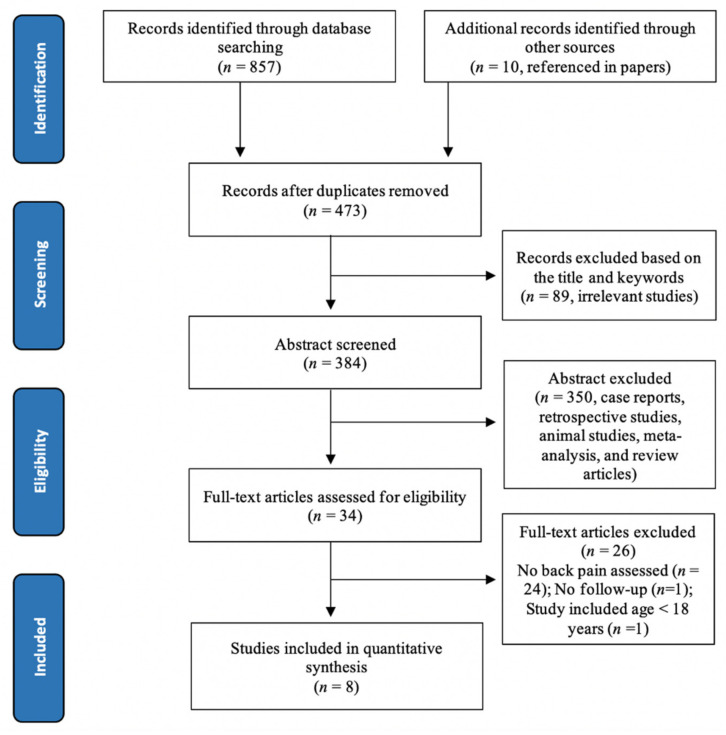
Flow chart showing the selection of articles for back pain evaluation after bariatric surgery in accordance with the Preferred Reporting Items for Systematic Reviews and Meta-analyses (PRISMA) guidelines.

The literature search is illustrated in the PRISMA flow diagram (Figure 1). Eight observational studies met the selection criteria for the purposes of the present review and were included in the final systematic review. All of the included studies consisted of eight cohort studies which were published from 2005 to 2019 [20,21,22,23,24,25,26,27].

### 3.2. Study Characteristics

Among a total of 298 patients in the eligible studies, the median of the mean age was 44.1 years, of which 75.8% were female. The mean overall follow-up was 42.4 weeks (range from 13 to 104.4 weeks).

The bariatric interventions in these studies included Roux-en-Y gastric bypass [20,21,22,25,26], sleeve gastrectomy [20,21,23,24,26], gastric banding [26,27] and duodenal switch [26]. Three studies reported that surgical interventions were performed laparoscopically [23,24,26], and six studies reported open interventions [20,21,22,25,26,27]. The study characteristics of all included studies are presented in Table 1.

### 3.3. Risk of Bias

The methodological quality of these cohort studies was assessed using the NOS-tools in Table 2. All studies were awarded more than seven stars, which demonstrated high-quality.

### 3.4. Back Pain Intensity

**Figure 2:**
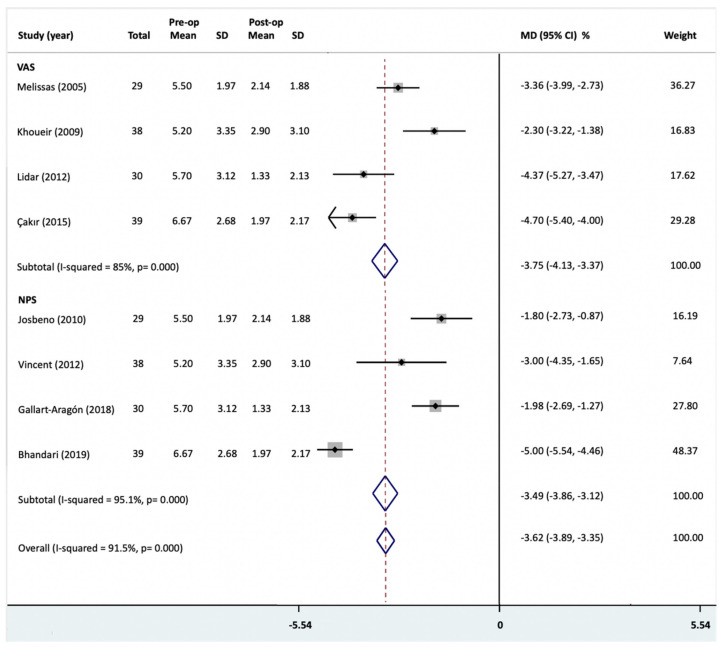
Back pain score changes following bariatric surgery were reported as mean difference (MD) and 95% confidence intervals (CI). Temporal analysis based on different pain score questionnaires was performed. VAS, Visual Analogue Scale; NPS, Numeric Rating Pain Scale; Pre-op, preoperative; Post-op, post-operative; SD, standard deviation; MD, mean difference.

All of the studies show a favorable improvement in back pain intensity scores (NPS and VAS) after bariatric surgery. Based on all of the eight included studies, the change in LBP after bariatric surgery showed a 40% to 86.7% reduction (MD = −3.62 (95% CI = −3.89, −3.35)) (Figure 2) [20,21,22,23,24,25,26,27].

We obtained subgroup analysis based on a different pain score questionnaire. Four studies measured pain intensity using NPS [20,22,24,25] and four studies measured pain intensity using VAS [21,23,26,27]. Among all patients, the mean back pain intensity score was 2.9 points and 3.8 cm lower after bariatric surgery compared to before in the NPS group and VAS group, respectively. In our meta-analysis, the change in NPS score and VAS score after bariatric surgery showed a significant change in the fixed effects model (NPS group: MD = −3.49 (95% CI = −3.86, −3.12); VAS group: MD = −3.75 (95% CI = −4.13, −3.37)) (Figure 2). We rated the quality of evidence as low due to inconsistency in findings and a lack of blinding in estimates.

A sensitivity analysis of the results is listed in Table 3. As there was significant heterogeneity, we obtained subgroup analysis based on publication date, number of patients and follow-up period. It showed no effect on the heterogeneity. A funnel plot of the results of included trials appeared to be asymmetrical (Supplemental Figure S1, no publication bias). A sensitivity analysis of the results showed the heterogeneity was caused by Bhandari et al. [20] in the NPS group and Khoueir et al. [21] in the VAS group. After re-analysis of the data, the results showed homogeneity (NPS group: MD = −2.07 (95% CI = −2.59, −1.55), *I*^2^ = 9.2%; VAS group: MD = −4.58 (95% CI = −5.13, −4.03), *I*^2^ = 0.0%).

### 3.5. BMI Change

**Figure 3:**
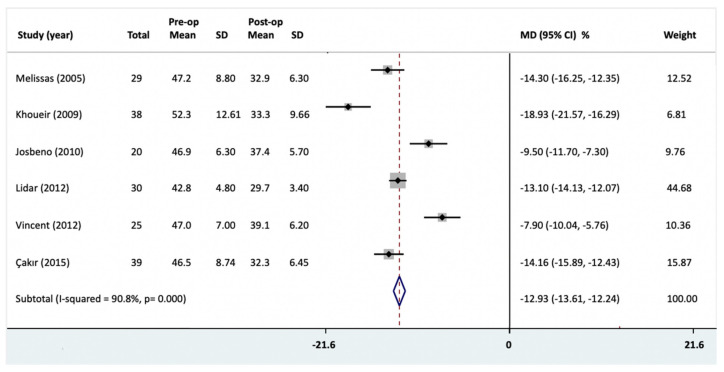
Body mass index (BMI) changes following bariatric surgery were reported as mean difference (MD) and 95% confidence intervals (CI). Pre-op, preoperative; Post-op, post-operative; SD, standard deviation; MD, mean difference.

Six studies reported the initial and post-operative BMI [21,22,23,25,26,27]. In a total of 181 patients from these six studies, the mean initial BMI was 47.1 kg/m^2^ (range from 42.8 to 52.25 kg/m^2^) and the mean reduction in BMI was 25.9% (ranged from 16.8% to 30.5%). There was a statistically significant reduction in mean BMI following bariatric surgery (Figure 3) (six studies [21,22,23,25,26,27]; MD = −12.93 (95% CI = −13.61, −12.24)). Heterogeneity was high and statistically significant (*I^2^* = 90.8%; *p* = 0.000). We rated the quality of evidence as moderate due to inconsistency in findings and the large magnitude of effect.

As there was significant heterogeneity, we obtained subgroup analysis based on publication date (95% CI = −0.56, 0.24; I^2^ = 84.5%), number of patients (95% CI = −0.27, 0.11; I^2^ = 83%) and follow-up period (95% CI = −0.22, 0.17; I^2^ = 89%). It showed no effect on the heterogeneity.

### 3.6. The Association of Mean Pain Change with BMI Change

Six studies reported BMI changes and pain intensity changes following bariatric surgery [21,22,23,25,26,27]. As shown in Table 3, no significant relationship between BMI change and decrease in clinical scores could be established (r = 0.44, (95% CI = −4.24, 5.12), *p* = 0.806). Based on different pain score questionnaires for subgroup analysis, no significant association was found between the change in BMI and decrease in clinical scores (Table 3).

### 3.7. Disability Change

**Figure 4:**
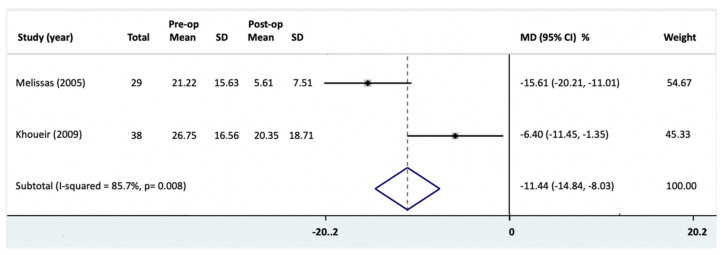
Oswestry Disability Index (ODI) changes following bariatric surgery were reported as mean difference (MD) and 95% confidence intervals (CI). Pre-op, preoperative; Post-op, post-operative; SD, standard deviation; MD, mean difference.

Three of the eight studies investigated the disability outcomes: (a) Roland–Morris disability questionnaire (RMD) [20,27], (b) ODI [21,27], and (c) Waddell disability index [27]. There was a statistically significant reduction in ODI following bariatric surgery by the mixed effect model (Figure 4, MD = −11.44 (95% CI = −14.84, −8.03)). Heterogeneity was high and statistically significant (*I^2^* = 85.7%; *p* = 0.008). The large magnitude of effect upgraded the low-quality evidence from the cohort studies to high-quality. However, inconsistency in findings downgraded the quality of statistically significant difference between the initial and post-operative BMI to moderate.

### 3.8. Other Clinical Outcomes

The intervertebral disc space height was examined by Lidar et al. in 30 morbidly obese patients undergoing bariatric surgery to determine the effect on axial and radicular pain, intervertebral disc space height, and quality of life one year after surgery [26]. One year post-operatively, the L4–L5 disc space height on CT scans significantly improved from 6 mm to 8 mm. The PPT was measured by Gallart-Aragón et al. in 72 morbidly obese patients undergoing sleeve gastroplasty to investigate quality of life and pain. No significant changes were seen in PPTs after SG [24].

## 4. Discussion

Here, we have performed meta-regression for the first time addressing the association of mean back pain changes with BMI change following bariatric surgery on obese patients. We identified a total of eight cohort studies with 298 obese patients who underwent bariatric surgery including Roux-en-Y gastric bypass, sleeve gastrectomy, gastric banding, and duodenal switch. All studies reported BMI and pain scores for back pain before and after bariatric surgery.

Results of our meta-analysis revealed that BMI in obese patients was statistically significantly lower following bariatric surgery across all eight included studies, which is consistent with previous recommendations on the use of bariatric surgery for obesity [28,29]. Meanwhile, our meta-analysis showed a significant decrease in back pain scores in obese patients following bariatric surgery over the eligible studies. Among all patients, the mean change in back pain score in NPS was 2.9 points and in VAS was 3.8 cm following bariatric surgery. These changes are equivalent to previous definitions of the minimal clinically important change in back pain intensity [30]. Moreover, an improvement in disability was reported in two of the studies based on ODI score following bariatric surgery [21,27]. Despite the plethora of research focused on gaining an understanding of the interplay between BMI change and LBP following bariatric surgery, many of the underlying mechanisms remain obscure. 

In exploring the causation of improved back pain following bariatric surgery, some authors have proposed many different etiological factors, such as mechanical change or musculoskeletal change and biochemical change [8,26,31,32,33]. Obesity can reduce the range of motion of the spine by postural adaptation and increase the mechanical load on the spine by causing a higher compressive force or increased shear stress on the lumbar spine [34]. As a consequence of the reduction in BMI following bariatric surgery, the mechanical loading on the intervertebral disc or facet joint is further reduced, which may cause the decrease in back pain. Furthermore, the low-grade systemic inflammation resulting from increased production of cytokines and acute-phase reactants in obese patients act as mediators for intervertebral disc degeneration, which might explain the effect of obesity on the back pain [31,32,33]. There is compelling evidence to indicate that obesity is strongly associated with LBP.

Weight loss following a healthy diet and effective physical activity is also likely to have a positive effect on back pain symptoms. Maintaining a healthy body weight may be one of the factors preventing the occurrence of LBP. Although all of the included studies reported a significant reduction in BMI and back pain score following bariatric surgery, there is still no consensus on the association between the reduction in BMI and the decrease in pain score [20,21,23,26]. In our meta-regression, there is no significant correlation between BMI change and back pain score change.

Although we did not find a direct linear correlation between the BMI reduction and the decrease in back pain following bariatric surgery, we think that this finding may reflect the major limitation of our study where the potentially included “population” (obese patients with back pain) studied were excluded for not reporting on back pain; hence, the true correlation of poor clinical results to BMI changes remains undetermined. Another possible explanation is that the lack of association may be explained by the observation that in severely obese patients, the disc and facet joints are exposed to many years of considerable axial loads, combined with normal aging and dehydration of the disc. These changes, in turn, may lead to BMI recovery, causing them to behave in a stepwise rather than in a linear fashion. These changes indicate that even if the BMI is reduced, the state of back pain by intervertebral disc degeneration will not change.

### Limitations of the Study

There are several limitations to this study that should be considered. Firstly, this meta-analysis only included prospective observational studies without control groups. The analysis resulted in too much heterogeneity between the pooled studies. A fix effect model showed a significant reduction. However, with the low-quality of the results, a definite conclusion cannot be drawn until more studies become available. Secondly, the range of follow-up period was large (from 3 to 24 months). There is a great heterogeneity in the time of data collection of the post test. Thirdly, the mean follow-up of the studies was short. No conclusion about long-term effects and the durability of the back pain problems can be made. Fourthly, the history of the patients’ back pain problems is unknown in most studies. Fifthly, meta-regression analysis describes observational associations across trials because comparisons of trial-level characteristics lack the benefit of randomization to support causal interpretation of findings. Consequently, associations between trial-level characteristics and effects of interventions are subject to the same limitations as findings from observational studies, such as bias by unmeasured confounding. Lastly, not all included studies reported the loss in patients follow-up. Still considering the limitations, more research is needed to establish if bariatric surgery should be considered prior to spinal surgery in morbidly obese patients solely with back pain.

## 5. Conclusions

From this meta-analysis, the data of back pain improvement following bariatric surgery are encouraging. However, considering the high heterogeneity, the evidence is of low quality. More research is needed to draw a correct weighted conclusion. Ideally, a prospective study including spinal imaging, inflammatory markers, longer follow-up periods, and larger study group with a randomized control group needs to be performed. The relationship between weight loss and reduction in back pain is complex but remains scientifically unclear. To further understand how and why bariatric surgery could reduce the back pain problems of obese patients, the mechanical and inflammatory effects of obesity on the spine should be better understood.

## Figures and Tables

**Table 1:** Demographic data, surgical technique, and surgery-related clinical outcomes for the selected studies.

Author_Year	Population				Outcomes						Follow-Up (Months)	Study Design
	N. Total	Intervention	N. Female	Mean Age (Years)	Initial BMI (kg/m^2^)	Post-Op BMI (kg/m^2^)	LBP	Pre-Op LBP	Post-Op LBP	Disability		
Melissas_2005	29	VBG	23	37.5	47.2 ± 8.8	32.9 ± 6.3	VAS	5.5 ± 1.97	2.14 ± 1.88	ODI/RMD/WDI	24	Cohort study
Khoueir_2009	38	RYGB or DS or SG	30	48.4	52.3 ± 12.61	38.32 ± 9.66	VAS	5.2 ± 3.35	2.9 ± 3.1	Yes/ODI	12	Cohort study
Josbeno_2010	20	LRYGB	18	41.6	46.9 ± 6.3	37.4 ± 5.7	NPS	3.5 ± 1.8	1.7 ± 2.63		3	Cohort study
Lidar_2012	30	LAGB or SG or LRYGB or DS	15	49	42.8 ± 4.8	29.7 ± 3.4	VAS	5.7 ± 3.12	1.33 ± 2.13		12	Cohort study
Vincent_2012	25	LRYGB or LAGB	20	41	47 ± 7	39.1	NPS	5.5 ± 4	2.5 ± 3.7		3	Cohort study
Çakır_2015	39	SG	39	37.7	46.49	32.3	VAS	6.67 ± 2.68	1.97 ± 2.17		6	Cohort study
Gallart-Aragón_2018	72	SG	47	45.36	-	-	NPS	3.95 ± 3.73	1.97 ± 2.95		6	Cohort study
Bhandari_2019	45	SG or one-anastomosis gastric bypass or LRYGB	34	54.7	54.2 ± 8.6	-	NPS	7.3 ± 1.4	2.3 ± 1.4	RMD	12	Cohort study

N., number; Pre-op, preoperative; Post-op, post-operative; BMI, body mass index; DS, duodenal switch gastric bypass; LAGB, laparoscopic adjustable gastric banding; LRYGB, Laparoscopic Roux-en-Y gastric bypass; RYGB, Roux-en-Y gastric bypass (open and laparoscopic); SG, sleeve gastroplasty; VBG, vertical banded gastroplasty; VAS, Visual Analogue Scale; NPS, Numeric Rating Pain Scale; ODI, Oswestry Disability Index; RMD, Roland–Morris disability questionnaire; WDI, Waddell disability index.

**Table 2:** Assessment of the methodological quality of the studies according to the Newcastle–Ottawa Scale (NOS).

Author	Year	Country	Surgical Procedures	Selection (/4)	Comparability (/2)	Outcome (/3)	Total (/9)
Melissas	2005	Greece	VBG	4	0	3	7
Khoueir	2009	USA	RYGB or DS or SG	4	0	3	7
Josbeno	2010	USA	LRYGB	4	0	3	7
Lidar	2012	Israel	LAGB or SG or LRYGB or DS	4	0	3	7
Vincent	2012	USA	LRYGB or LAGB	4	1	3	8
Çakır	2015	Turkey	SG	4	0	3	7
Gallart-Aragón	2018	Spain	SG	4	0	3	7
Bhandari	2019	India	SG or one-anastomosis gastric bypass or LRYGB	4	0	3	7

Note: A study awarded seven or more stars is regarded as a high-quality study.

**Table 3:** Grading of Recommendations Assessment, Development and Evaluation (GRADE) level of quality assessment. The table presents a detailed summary of the evidence, including statistical model (mean difference and associated confidence intervals (CI)), regression data, tests of homogeneity, publication bias (Begg’s test), and the certainty of the evidence.

Outcome	Test	Statistical Model			Homogeneity		Begg’s P	Level of Quality
		MD/r	95% CI	*p* Value	*p* Value	*I*^2^ (%)		
NPS	Meta-analysis	−3.49	−3.86, −3.12 ^a^	-	0.000 ***^,b^	95.1 ^c^	1.000	L ^1,2,3^
	Sensitivity analysis				-			
	Publication date	-	−0.68, 1.06	-		94.8	-	
	Follow-up period		−0.24, 0.86			76.1		
	Number of patients		−0.19, 0.20			96.7		
	Meta-regression with BMI	-						
VAS	Meta-analysis	−3.75	−4.13, −3.37 ^a^	-	0.000 ***^,b^	85 ^c^	0.12	L ^1,2,3^
	Sensitivity analysis				-			-
	Publication date	-	−2.46, 1.68	-		60.8	-	
	Follow-up period		−3.52, 3.97			90		
	Number of patients		−3.14, 3.29			92.4		
	Meta-regression with BMI	1.87	−1.83, 5.57	0.162	-			
ODI	Meta-analysis	−11.44	−14.84, −8.03 ^a^	-	0.008 **^,b^	85.7 ^c^	1.000	M ^1,2,3^
	Sensitivity analysis							
	Publication date	-						
	Follow-up period							
	Number of patients							
	Meta-regression with BMI							

Pre-op, preoperative; post-op, post-operative; CI, confidence intervals; MD, mean difference; VAS, Visual Analogue Scale; NPS, Numeric Rating Pain Scale; ODI, Oswestry Disability Index. ^a^ 95% CI including 0 means no statistical significance, while not including 1 means have statistical significance; ^b^ *p* < 0.05 indicated significance; ^c^ *I^2^* > 50% implied heterogeneity. Quality of evidence: H = high, M = moderate, L = low, VL = very low. Significant difference, ** *p* < 0.01, *** *p* < 0.001. ^1^—rated down for risk of bias; ^2^—rated down for inconsistency; ^3^—rated up for large magnitude of effect (strong evidence of association—significant relative risk of >2 (<0.5) based on consistent evidence from two or more observational studies, with no plausible confounders (+1); very strong evidence of association—significant relative risk of >5 (<0.2) based on direct evidence with no major threats to validity (+2)).

## Data Availability

Not applicable.

## Supplementary Materials

The following are available online at https://www.mdpi.com/article/10.3390/jcm10071443/s1. The search strategy for each database is available in the Supplemental Table S1. The details of Grading of Recommendations Assessment, Development and Evaluation (GRADE) system were list in Supplemental Table S2. The funnel plot for publication bias (Inverse Variance method) in Supplemental Figure S1.

## References

[1] World Health Organization (2016). Obesity and Overweight: Fact Sheet.

[2] Goodwin P.J., Stambolic V. (2015). Impact of the obesity epidemic on cancer. Annu. Rev. Med..

[3] Guh D.P., Zhang W., Bansback N., Amarsi Z., Birmingham C.L., Anis A.H. (2009). The incidence of co-morbidities related to obesity and overweight: A systematic review and meta-analysis. BMC Public Health.

[4] Haslam D.W., James W.P.T. (2005). Obesity. Lancet.

[5] Atchison J.W., Vincent H.K. (2012). Obesity and low back pain: Relationships and treatment. Pain Manag..

[6] Shiri R., Karppinen J., Leino-Arjas P., Solovieva S., Viikari-Juntura E. (2009). The association between obesity and low back pain: A meta-analysis. Am. J. Epidemiol..

[7] Liuke M., Solovieva S., Lamminen A., Luoma K., Leino-Arjas P., Luukkonen R., Riihimäki H. (2005). Disc degeneration of the lumbar spine in relation to overweight. Int. J. Obes..

[8] Samartzis D., Karppinen J., Chan D., Luk K.D.K., Cheung K.M.C. (2012). The association of lumbar intervertebral disc degeneration on magnetic resonance imaging with body mass index in overweight and obese adults: A population-based study. Arthritis Rheum..

[9] Urquhart D.M., Berry P., Wluka A.E., Strauss B.J., Wang Y., Proietto J., Jones G., Dixon J.B., Cicuttini F.M. (2011). 2011 young investigator award winner. Spine.

[10] Urquhart D.M., Kurniadi I., Triangto K., Wang Y., Wluka A.E., O’Sullivan R., Jones G., Cicuttini F.M. (2014). Obesity is associated with reduced disc height in the lumbar spine but not at the lumbosacral junction. Spine.

[11] Robinson M.K. (2009). Surgical treatment of obesity—Weighing the facts. N. Engl. J. Med..

[12] Arterburn D., Wellman R., Emiliano A., Smith S.R., Odegaard A.O., Murali S., Williams N., Coleman K.J., Courcoulas A., Coley R.Y. (2018). Comparative effectiveness and safety of bariatric procedures for weight loss. Ann. Intern. Med..

[13] Joaquim A.F., Helvie P., Patel A.A. (2019). Bariatric surgery and low back pain: A systematic literature review. Glob. Spine J..

[14] Stefanova I., Currie A.C., Newton R.C., Albon L., Slater G., Hawkins W., Pring C. (2020). A meta-analysis of the impact of bariatric surgery on back pain. Obes. Surg..

[15] Moher D., Liberati A., Tetzlaff J., Altman D.G., The PRISMA Group (2009). Preferred reporting items for systematic reviews and meta-analyses: The PRISMA statement. PLoS Med..

[16] Wells G., Shea B., O’Connel D., Robertson J., Peterson J., Welch V., Losos M., Tugwell P. (2000). The Newcastle-Ottawa Scale (NOS) for Assessing the Quality of Nonrandomised Studies in Meta-Analyses.

[17] Moher D., Cook D.J., Eastwood S., Olkin I., Rennie D., Stroup D.F. (1999). Improving the quality of reports of meta-analyses of randomised controlled trials: The quorom statement. Lancet.

[18] Stroup D.F., Berlin J.A., Morton S.C., Olkin I., Williamson G.D., Rennie D., Moher D., Becker B.J., Sipe T.A., Thacker S.B. (2000). Meta-analysis of observational studies in epidemiologya proposal for reporting. JAMA.

[19] Balshem H., Helfand M., Schünemann H.J., Oxman A.D., Kunz R., Brozek J., Vist G.E., Falck-Ytter Y., Meerpohl J.J., Norris S.L. (2011). Grade guidelines: 3. rating the quality of evidence. J. Clin. Epidemiol..

[20] Bhandari M., Mathur W., Kosta S., Salvi P., Fobi M. (2019). Assessment of functional ability of nonambulatory patients with obesity: After and before bariatric surgery. Surg. Obes. Relat. Dis..

[21] Khoueir P., Black M.H., Crookes P.F., Kaufman H.S., Katkhouda N., Wang M.Y. (2009). Prospective assessment of axial back pain symptoms before and after bariatric weight reduction surgery. Spine J..

[22] Vincent H.K., Ben-David K., Conrad B.P., Lamb K.M., Seay A.N., Vincent K.R. (2012). Rapid changes in gait, musculoskeletal pain, and quality of life after bariatric surgery. Surg. Obes. Relat. Dis..

[23] Çakır T., Oruç M.T., Aslaner A., Duygun F., Yardımcı E.C., Mayir B., Bülbüller N. (2015). The effects of laparoscopic sleeve gastrectomy on head, neck, shoulder, low back and knee pain of female patients. Int. J. Clin. Exp. Med..

[24] Gallart-Aragón T., Fernández-Lao C., Galiano-Castillo N., Cantarero-Villanueva I., Lozano-Lozano M., Arroyo-Morales M. (2018). Improvements in health-related quality of life and pain: A cohort study in obese patients after laparoscopic sleeve gastrectomy. J. Laparoendosc. Adv. Surg. Tech..

[25] Josbeno D.A., Jakicic J.M., Hergenroeder A., Eid G.M. (2010). Physical activity and physical function changes in obese individuals after gastric bypass surgery. Surg. Obes. Relat. Dis..

[26] Lidar Z., Behrbalk E., Regev G.J., Salame K., Keynan O., Schweiger C., Appelbaum L., Levy Y., Keidar A. (2012). Intervertebral disc height changes after weight reduction in morbidly obese patients and its effect on quality of life and radicular and low back pain. Spine.

[27] Melissas J., Kontakis G., Volakakis E., Tsepetis T., Alegakis A., Hadjipavlou A. (2005). The effect of surgical weight reduction on functional status in morbidly obese patients with low back pain. Obes. Surg..

[28] Padwal R., Klarenbach S., Wiebe N., Birch D., Karmali S., Manns B., Hazel M., Sharma A.M., Tonelli M. (2011). Bariatric surgery: A systematic review and network meta-analysis of randomized trials. Obes. Rev..

[29] Buchwald H., Avidor Y., Braunwald E., Jensen M.D., Pories W., Fahrbach K., Schoelles K. (2004). Bariatric surgery: A systematic review and meta-analysis. JAMA.

[30] Myles P.S., Myles D.B., Galagher W., Boyd D., Chew C., MacDonald N., Dennis A. (2017). Measuring acute postoperative pain using the visual analog scale: The minimal clinically important difference and patient acceptable symptom state. Br. J. Anaesth..

[31] Risbud M.V., Shapiro I.M. (2014). Role of cytokines in intervertebral disc degeneration: Pain and disc content. Nat. Rev. Rheumatol..

[32] Wuertz K., Haglund L. (2013). Inflammatory mediators in intervertebral disk degeneration and discogenic pain. Glob. Spine J..

[33] Podichetty V.K. (2007). The aging spine: The role of inflammatory mediators in intervertebral disc degeneration. Cell. Mol. Boil..

[34] Vismara L., Menegoni F., Zaina F., Galli M., Negrini S., Capodaglio P. (2010). Effect of obesity and low back pain on spinal mobility: A cross sectional study in women. J. Neuroeng. Rehabil..

