# Supplementary material for: Changes in Back Pain Scores after Bariatric Surgery in Obese Patients: A Systematic Review and Meta-Analysis
Source: J Clin Med. 2021 Apr 1;10(7):1443. doi: 10.3390/jcm10071443 (PMC8036450; doi:10.3390/jcm10071443)

Supplemental Table S1. Database formulas during the literature search.

Database	Bariatric surgery terms		Back pain terms	Search strategy	Results
Pubmed	(((("Gastric Bypass"[Mesh]) OR ("Gastrectomy"[Mesh]) OR ("Bariatrics"[Mesh]) OR ("Biliopancreatic Diversion"[Mesh]) OR ("Gastroplasty"[Mesh]) OR ("Jejunoileal Bypass"[Mesh]) OR ("Bariatric Surgery"[Mesh] OR "bariatric surgery" OR "bariatric surgeries" OR "roux-en-y gastric bypass" OR "greenville gastric bypass" OR "gastroileal bypass" OR gastrojejunostomy OR gastrojejunostomies OR gastroplasties OR collis gastroplasty OR vertical-banded gastroplasty OR "vertical banded gastroplasty" OR "vertical banded gastro plas" OR "jejunoileal bypass" OR "ileojejunal bypasses" OR "intestinal bypass" OR "intestinal bypasses" OR "biliopancreatic bypass" OR "biliopancreatic diversion" OR "duodenal switch" OR "pancreatobiliary bypass" OR "gastric banding" OR "stomach banding" OR "swedish gastric banding" OR" swedish adjustable gastric banding" OR "laparoscopic adjustable gastric banding" OR "laparoscopic adjustable silicone banding" OR "bariatric operation" OR "bariatric operations" OR "bariatric procedure" OR "bariatric procedures" OR "bariatric surgical procedure" OR "bariatric surgical procedures" OR "obesity surgery" OR "sleeve gastrectomy" OR "gastric sleeve" OR "lrygb" OR "laparoscopic sleeve gastrectomy" OR SG OR LSG OR "bariatrics" OR "bariatric" OR "metabolic surgery" OR "weight loss surgery" OR GB OR "biliopancreatic diversion" OR "duodenal switch" OR "omega switch" OR "sleeve resection" OR "duodenojejunal bypass" OR "jejunoileal bypass" OR "jejuno-ileal bypasses" OR "ileojejunal bypass"))))	AND	((back pain[MeSH terms]) or low back pain[MeSH terms] or back pain* or lumb* pain or lumbago or backache* or back ache* OR "backpain" OR "back pain" OR "Back ache" OR backache OR "lower back pain" OR "Low-back pain" OR "Low back pain" OR "back disorder" OR lumbago OR "lumbar pain" OR Sciatica OR Vertebrogenic Pain Syndrom* OR "musculoskeletal pain")))	#1 AND #2<br>Sort by: **Best Match**<br>Filters: **English**	➔ 408 articles<br>➔ Title screening: 45 articles<br>➔ Abstract screening: 26<br>➔ Full text 12
MEDLINE	Bariatrics/ OR Bariatric Surgery/ OR Biliopancreatic Diversion/ OR Gastrectomy/ OR Gastric Bypass/ OR Gastroplasty/ OR Jejunoileal Bypass/ OR Anastomosis, Roux-en-Y/	AND	Back Pain/ OR<br><br>Low Back Pain/ OR Intervertebral Disc Displacement/ OR Sciatica/ OR<br><br>Spinal Diseases/ OR Musculoskeletal Pain/	Filter: English	➔ **36** studies<br>➔ **7** fitting, 5 maybe (all double)<br>➔ **6** abstract screening<br>➔ **3** fitting full text (double)
EMBASE	Bariatric surgery OR bariatric*.mp. OR stomach bypass OR Roux Y anastomosis OR Roux-en-Y gastric bypass OR gastroplasty OR gastric bypass surgery OR jejunoileal bypass OR intestine bypass OR biliopancreatic bypass OR gastric banding OR sleeve gastrectomy OR gastric sleeve OR laparoscopic sleeve gastrectomy	AND	Low back pain OR back pain*.mp. OR backache OR spine disease OR sciatica OR musculoskeletal pain	Filter: English	➔ 413 articles in total<br>➔ **13** abstracts<br>➔ **8** eligible full text screening (double)
CRCC	Bariatrics/ OR Bariatric Surgery/ OR Biliopancreatic Diversion/ OR Gastrectomy/ OR Gastric Bypass/ OR Gastroplasty/ OR Jejunoileal Bypass/ OR Anastomosis, Roux-en-Y/	AND	Back Pain/ OR<br><br>Low Back Pain/ OR Intervertebral Disc Displacement/ OR Sciatica/ OR<br><br>Spinal Diseases/ OR	Filter: English	-

			Musculoskeletal Pain/		
Other resources (Google scholar and references)	-	-	-	Filter: English	10 paper from references

Supplemental Table S2. Grading of Recommendations Assessment, Development and Evaluation (GRADE) approach for rating the quality of estimates of treatment effect

GRADE Assessment
**Ratings**<br>**High quality** (⊕⊕⊕⊕)—We are very confident that the true effect lies close to that of the estimate of the effect<br>**Moderate quality** (⊕⊕⊕O)— We are moderately confident in the effect estimate: The true effect is likely to be close to the estimate of the effect, but there is a possibility that it is substantially different<br>**Low quality** (⊕⊕OO)— Our confidence in the effect estimates is limited: The true effect may be substantially different from the estimate of the effect<br>**Very low quality** (⊕OOO)— We have very little confidence in the effect estimate: The true effect is likely to be substantially different from the estimate of effect
**Down rating**<br>The quality rating may be rated down by −1 (serious concern) or −2 (very serious concern) for the following reasons<br>• Risk of bias (such as failure to conceal random allocation or blind participants in randomised controlled trials or failure to adequately control for confounding in observational studies)<br>• Inconsistency (such as heterogeneity of estimates of effects across trials)<br>• Indirectness (such as surrogate outcomes, study populations or interventions that differ from those of interest, or intransitivity)<br>• Imprecision (for example, 95% confidence intervals are wide and include or are close to null effect)<br>• Publication bias
**Up rating**<br>Rating up is typically applied only to observational studies; the most common reason is for a large or very large effect seen over a short period of time and altering a clear downward trajectory

In the GRADE approach, RCTs start as high-quality evidence and cohort studies as low-quality evidence.

Supplemental Figure S1. Funnel plot for publication bias

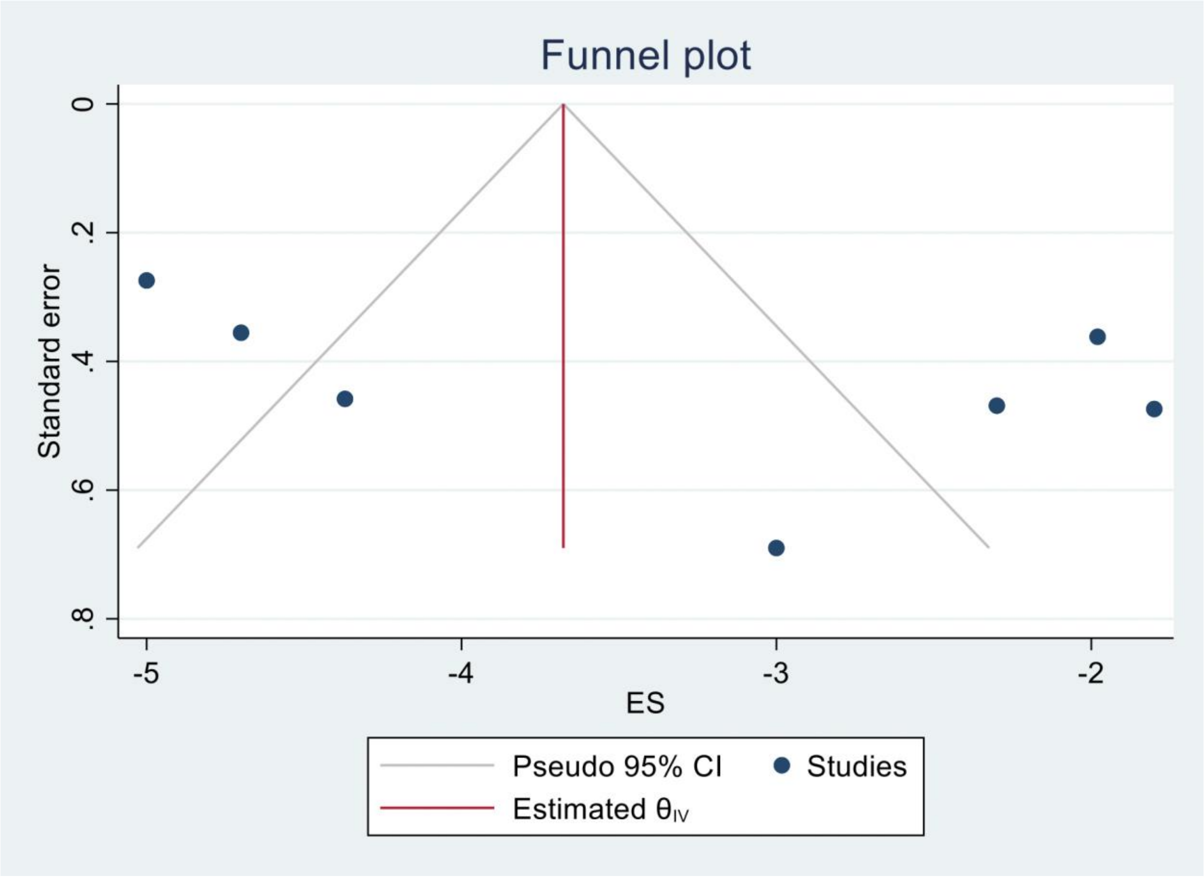

Supplement: Supplementary file 1 [file jcm-10-01443-s001.pdf]
